# Circulatory miR-34a as an RNA-based, noninvasive biomarker for brain aging

**DOI:** 10.18632/aging.100371

**Published:** 2011-10-16

**Authors:** Xiaoli Li, Amit Khanna, Na Li, Eugenia Wang

**Affiliations:** ^1^ Department of Biochemistry and Molecular Biology, University of Louisville School of Medicine, Louisville, Kentucky, USA; ^2^ Gheens Center on Aging, University of Louisville School of Medicine, Louisville, Kentucky, USA

**Keywords:** MicroRNAs, aging, biomarker, SIRT1, Bcl2, miR-34a, miR-196a

## Abstract

MicroRNAs in blood samples have been identified as an important class of biomarkers, which can reflect physiological changes from cancer to brain dysfunction. In this report we identify concordant increases in levels of expression of miR-34a in brain and two components of mouse blood samples, peripheral blood mononuclear cells (PBMCs) and plasma, from 2 day old neonates through young adulthood and mid-life to old age at 25 months. Levels of this microRNA's prime target, silent information regulator 1 (SIRT1), in brain and the two blood-derived specimens decrease with age inversely to miR-34a, starting as early as 4 months old, when appreciable tissue aging has not yet begun. Our results suggest that: 1. Increased miR-34a and the reciprocal decrease of its target, SIRT1, in blood specimens are the accessible biomarkers for age-dependent changes in brain; and 2. these changes are predictors of impending decline in brain function, as early as in young adult mice.

## INTRODUCTION

The list of genetic factors pertinent to life-span determination has been growing by leaps and bounds, to define age-related physiological changes [1]. A steady loss of function in multiple vital organs has been shown to correlate with aging, accompanied by increased incidence of a wide range of diseases such as neurological disorders, metabolic disorders *e.g.* diabetes, and cancer [2]. Modification of signaling cascades, such as highly conserved signaling pathways including insulin/Insulin-like growth factor 1 (IGF1), target of rapamycin (TOR) and sirtuin, for enhanced cellular responses to stress, is recognized as key to life span extension and accompanying reduced age-related pathology [3]. The search for biomarkers for aging has led to the identification of unique leads in blood samples, a relatively noninvasive method to acquire experimental specimens. However, the main findings in circulating blood reflecting systemic phenomena are protein-based changes, mostly connected to agerelated perils such as cancer, atherosclerosis, and cardiovascular dysfunction [4]. A few studies linking blood-based changes to normal aging involve a serum protein pattern that proves to be a reliable index for aging in rat, independent of pathologies [5]; the techniques commonly used, 2-D gel electrophoresis or proteomic profiling, have led to identifying various proteins as potent blood-based biomarkers [6]. Another such example is the observation that circulating IGF-1 in centenarians with robust cognition is low [7], while those suffering Alzheimer's disease (AD) show high levels of this protein in their blood [8].

Non-coding RNAs are prominent epigenetic factors, along with nucleic acid modifications such as DNA methylation and histone/chromatin modification [9]. Among all the small non-coding RNAs, microRNAs (miRNAs) have been studied in detail; they are generally transcribed by RNA polymerase II, rarely by RNA polymerase III [10]. MiRNAs modulate protein regulation at the post-transcriptional level, because their seed sequences have perfect or partial complementarity to the coding region or the 3'- untranslated region (UTR) of one or more target mRNAs, leading to mRNA degradation in the former case, and inhibition of protein translation in the latter; either way, they serve as negative regulators of gene expression [10, 11]. Early studies illustrating the role of miRNAs in aging found that expression of C. elegans lineage 4 (lin-4) miRNA is needed for extended lifespan, while abridged expression leads to shortened lifespan [11, 12]. We also reported a set of miRNA expressions unique to peripheral blood mononuclear cells (PBMCs) of AD victims [13], as well as changes in miRNA expression during aging in brain and liver of mice and rats, including long-lived calorie-restricted and mutant mice with extended life span [14-17].

In general, blood samples can be separated into two major components: PBMCs, composed of lymphocytes, monocytes, megakaryocytes, platelets, etc.; and plasma or serum, depending on blood- collecting procedures (adding an anticoagulant produces the former instead of the latter). Our group has shown that miRNA profiles in PBMCs of AD victims differ from those of normal elderly controls (NEC); this led us to suggest that lead microRNAs in PBMCs of AD victims may be biomarkers as a blood-based diagnostic for this disease [18]. In cell-free serum/plasma, microRNAs are repeatedly reported to be not only present [19], but also a means for cell-cell communication, secretion, and many other cellular functions associated with cell death, etc. [5, 20, 21]. Recently, major differences in miRNA expression between plasma microvesicles and PBMCs were reported, owing to the origin of these cell-free miRNA expressions in the former case [20]. Plasma miRNA expression is a predictive and diagnostic tool for lung cancer; changes in plasma miRNA expression imply staging and prognosis [22]. Blood associated microvesicles contain miRNAs suggested for inter-cellular and inter-organ communication [23]. In brain, circulating microRNAs have been suggested to be vital for neuronal communication [24], and lead microRNAs in blood serve as circulating biomarkers for bipolar disorder and early Huntington's disease [25].

In this study we report that miR-34a levels in mouse PBMCs and plasma increase with age, as do those in brain, starting at 4 months of age, as documented in samples from 2 days old till 25 months of age. Parallel study of miR-196a shows that the level of this microRNA remains stable with age in all three specimens examined, and therefore serves as a control for age-independent regulation of its expression. Corresponding to this increase of miR-34a, plasma expression of SIRT1, the major target of this microRNA, shows a precipitous reciprocal decrease starting at 4 months of age, while PBMCs exhibit a gradual decrease in SIRT1 from this age onward. In contrast, the SIRT1 level in brain rises from 20 days old till 4 months, and remains at this level without decrease until 12 months of age. The reciprocal expression between miR-34a and SIRT1 in plasma samples is not observed with another target of this microRNA, Bcl2, whose expression in plasma actually declines as early as 7 days after birth and continues to 25 months, while in brain and PBMCs it shows a slight decrease during the same time frame. In brief, our results show here that levels of miR-34a in plasma and PBMCs may serve as a non- invasive biomarker, a circulating ‘footprint’ of brain aging; in particular the rise in the plasma is detected as early as 4 months of age, before any impending decline in this organ begins.

## RESULTS

### Expression levels of miRNAs in blood and brain samples of C57/B6 mice

Mouse microRNA 34a (mmu-miR-34a) expression increases in brain of older rodents, while a decline in its expression over age is linked to longevity in calorie-restricted mouse brain [13, 16]. SIRT1 and Bcl2 are two major target genes suppressed by miR-34a [17, 26]. In contrast, although miR-196a is elevated in Crohn's disease [27], changes in its level of expression during mouse aging is not yet reported. Thus, our study of microRNA expression over a life span from neonatal to old age was performed with miR-34a as our focus of interest, and miR-196a as a control.

**Figure 1:**
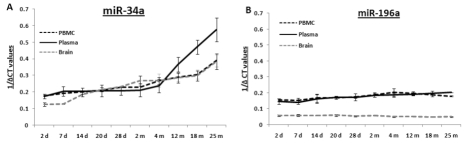
Age-dependent expression levels of two miRNAs in blood and tissue samples of C57/B6 mice A graphical representation of expression levels of miRNAs using qualitative PCR, represented as 1/ΔCt values as box plots for brain, PBMC and plasma samples. Age groups included are from early to old age [from 2 days (d) to 25 months (m)]. Panel **(A)** shows composite graphs for expression of miR-34a, while panel **(B)** shows the levels of expression of miR-196a in brain, PBMCs, and plasma. (n = 3; three different animals were used from each age group selected for the study.)

Total RNA samples extracted from PBMCs, plasma and brain specimens from age groups from 2 days to 25 months were reverse transcribed for quantitative PCR (qPCR) to determine their levels of mmu-miR-34a and mmu-miR-196a. qPCR for these two miRNAs was performed using total RNA samples from all three specimens (Supplemental Figure 1-2); composite graphs of expression of these two miRNAs are shown in Figure 1. Levels of miR-34a are stable from 2 days till 4 months, with subsequent rises in all three tissues; the increases in brain and PBMCs exhibit a similar gradual trend, while the increase in plasma samples rises steeply starting at 4 months. All assays for each animal were repeated three times, and three animals were used per age group; thus, the qPCR results presented here were calculated from nine data points, with three repeats of three different animals. In contrast to the increased miR-34a expression with age, levels of miR-196a remain stable throughout all age groups studied here, from 2 days till 25 months of age. Statistical analysis for each age group is presented in Supplemental Table 1.

### Inverse levels of expression between miR-34a and its target, SIRT1, in blood and brain during aging

**Figure 2:**
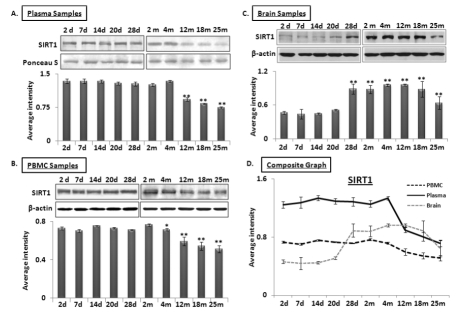
Age-dependent expression levels of SIRT1 in blood and tissue samples of C57/B6 mice Western blot analysis of SIRT1 expression in age groups from 2 days (d) to 25 months (m); panels **(A)** SIRT1 expression in plasma samples normalized with selected Ponceau S stained band, showing constant levels for all samples used, along with histograms presenting average densitometric values, **(B)** SIRT1 expression in PBMC samples normalized with β-actin, along with histograms presenting average densitometric values, **(C)** SIRT1 expression in brain samples normalized with β-actin, along with histograms presenting average densitometric values, **(D)** Composite graph presenting SIRT1 expression in plasma, PBMC and brain samples. (*p < 0.01, **p < 0.0001; all histograms represent Mean ± SD; n = 3; three different mice used from each selected age group.)

MicroRNA-34a regulates SIRT1 expression both at the pre-transcriptional level, by regulating expression of transcription factor SP1 [16], and also at the post-transcriptional level, by binding to the 3’ untranslated region (UTR) of SIRT1 transcript [28] to repress its expression. Total protein samples extracted from plasma, PBMCs, and brain were subjected to Western blot analysis to determine a possible inverse relationship between SIRT1 and miR-34a levels from 2 days to 25 months old. Decreased SIRT1 expression was observed in both plasma and PBMC samples at 4 months of age, with the former showing a precipitous drop in SIRT1 abundance (Figure 2A & B). However, levels of SIRT1 in brain increase from a steady state during the postnatal period to a stable high level from 28 days to 12 months, before decreasing to 25 months (Figure 2C). A composite graph depicting the expression trends in the three specimens across all age groups shows the precipitous decline in plasma, but gradual decline in PBMCs from 4 months on, and the brain-specific rise at 28 days followed by the decline at 12 months old (Figure 2D). In all, the only sharp inverse relationship detected at early adult life (4-months) between miR-34a and its target, SIRT1, is observed in plasma. All immunoblots were further verified by densitometric measurements of three repeats with three different animals of the same age group, after normalization with β-actin in brain and PBMC samples. Ponceau S stained bands were used to validate equal loading, and selected bands showing consistent levels across the blots were used to normalize SIRT1 expression in plasma samples. The individual graphs may be found as supplemental data (Supplemental Figure 3).

### Expression of miRNA-34a and its target SIRT1 in brain tissue sections from middle and old age C57/B6 mice

**Figure 3:**
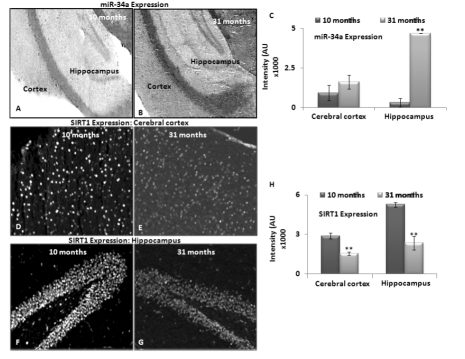
Expression of miRNA-34a and SIRT1 in brain tissue sections from middle and old age C57/B6 mice *In situ* hybridization detected miR-34a expression using LNA probes. Panels **(A)** and **(B)** show expression of miR-34a in cortex and hippocampus of 10 and 31 month old mice. Scrambled LNA probes were used as control for this experiment (Image not shown); **(C)** graphical presentation of mean intensity values of miR-34a expression in cortex and hippocampus of 10 and 31 month old mice, by immunostaining assay to detect SIRT1 expression. Panels **(D-E)** and **(F-G)** show expression of SIRT1 in cortex and hippocampus of 10 and 31 month old mice, by DAPI staining to detect nucleus and section integrity (Image not shown); **(H)** graphical presentation of mean intensity values of SIRT1 expression in cortex and hippocampus of 10 and 31 month old mice. (*p < 0.01, **p < 0.0001; all histograms represent Mean ± SD; n = 3; three different biological samples from each age group.)

A lack of age-dependent increase in miR-34a expression in the hippocampal region has been previously reported in calorie-restricted mice, as compared to littermate wild type (WT) controls [17]. Here, we performed similar in situ histochemical (ISH) determination of miR-34a, to validate the qPCR results with total brain RNA studies by locked nucleic acid (LNA) probes for this microRNA. Two selected age groups, 10 and 31 months, were chosen to represent specimens before SIRT1 decreases, and beyond the maximal decline. Brain sections of these two age groups were processed for levels of miR-34a expression by in situ hybridization, binding with a locked nucleic acid probe for miR-34a, as well as a control probe for background binding reaction (Figure 3 A & B).

Intensity of LNA probe reaction was most noted in the hippocampus, more than the cortex, in both age groups. This intensity differential was further verified by densitometric measurements of three serial sections each from three different animals of the same age groups (Figure 3C). Expression data obtained using in situ hybridization confirmed the qPCR results, and validated the trend of increased miR-34a expression from 10 to 31 months of age in brain.

Immunostaining to detect SIRT1 in middle and old age, i.e. 10 and 31 months respectively, was performed on sister specimens of those used for in situ hybridization with the LNA probe for miR-34a. Decreased SIRT1 from 10 to 31 months was observed most significantly in hippocampus, validating the inverse relationship with miR34a expression in brain, described by the grind-and-find qPCR assays and Western blotting for SIRT1 levels. Figure 3D-G shows in situ hybridization results of heightened miR-34a expression in hippocampus, corresponding to decreased SIRT1 presence. All immunostaining results were further verified by densitometric measurements of three repeats with three different animals (n = 3) (Figure 3 C, H).

### Levels of Bcl-2, another target of miR-34a, in blood and tissue samples of C57/B6 mice

**Figure 4:**
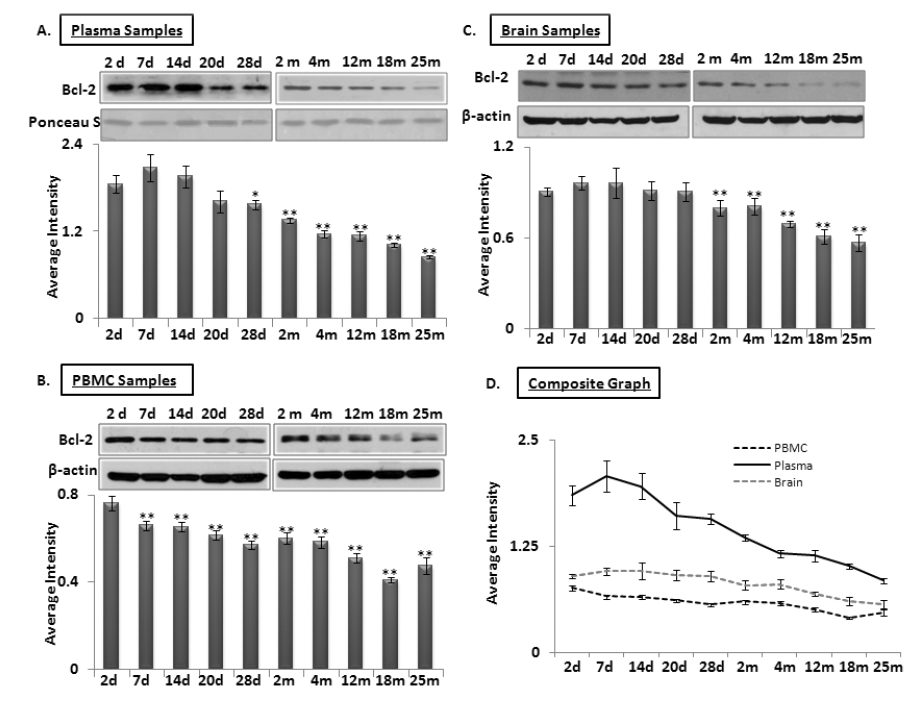
Age-dependent expression levels of Bcl-2 in blood and brain tissue samples of C57/B6 mice Western blot analysis for Bcl-2 levels in age groups from 2 days (d) to 25 months (m); panels **(A-B-C)** Bcl-2 expression normalized with Ponceau S stained bands in plasma, and with β-actin in brain and PBMC samples, along with histograms presenting average Bcl-2 expression densitometric values; **(D)** Composite graph presenting Bcl-2 expression in plasma, PBMC and brain samples as average densitometric intensity. All graphs represent Mean ± SD. n = 3; three different mice from each age group.

Another target of interest of miR-34a is Bcl2. Western blots for 26 kilodalton Bcl2 expression were performed using protein samples of brain, PBMCs and plasma from all age groups used for the qPCR assays of the two microRNAs. A decline in Bcl2 was observed starting as early as 7 days after birth in plasma (Figure 4 A-C), continuing until old age at 25 months. In contrast, Bcl2 in brain and PBMCs show stable abundance, with a gradual decrease from 2 months to old age. A composite graph showing the decline in Bcl2 is shown in Figure 4D; the individual graphs may be found as supplemental data (Supplemental Figure 4). Interestingly, the decrease of Bcl2 in plasma starts at 7 neonatal days, and continues its downward trend in plasma samples. All immunoblot data were verified by densitometric measurements of three repeats with three different animals, after normalization with β-actin for tissues and PBMC samples. For normalization of Bcl2 expression in plasma samples, Ponceau S stained bands were used to confirm equal loading (n = 3).

### Age-dependent changes of levels of p53 and its acetylated form in blood and brain samples of C57/B6 mice

**Figure 5:**
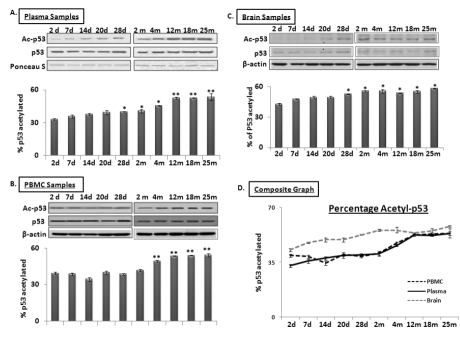
Age-dependent changes in ratio of acetylated to total p53 in blood and brain samples of C57/B6 mice Western blot analysis of acetylated p53 proportion in age groups from 2 days to 25 months; **Panels A, B, and C** show acetylated P53 proportion normalized with Ponceau S in plasma (Panel A), and with β-actin in PBMCs and brain samples, along with histograms presenting the average densitometric values. **Panel D** presents a composite graph of the percentage of acetylated P53 expression in the total p53 protein pool in plasma, PBMC and brain samples. (*p < 0.01, **p < 0.0001; all histograms represent Mean ± SD; n = 3; three different biological samples from each age group)

SIRT1 is an evolutionarily conserved molecule with deacetylation properties; one of its targets is p53, whose acetylation leads to increased transcriptional activation of miR-34a expression [29]. Western blot analysis was performed using total protein samples from PBMCs, plasma and brain from mice aged 2 days to 25 months. Expression of p53 and its acetylated form was detected; Ponceau S stained bands for plasma samples and β-actin for PBMCs and brain were used as normalization controls (n = 3). Figure 5 shows levels of total and acetyl-p53 in plasma (panel 5A), PBMCs (panel 5B) and brain (panel 5C), with the percentage of the latter in the total protein pool illustrated in Panel 5D. The acetyl-p53 proportion remains stable from 2 days until 2 months in both plasma and PBMC specimens; this is followed by a steady increase between 4 and 12 months, after which it stays the same in the remaining old age groups, as observed in brain. The acetyl-p53 fraction in brain exhibits a steady increase from 2 days old till old age. Interestingly, proportions of acetyl-p53 in the total p53 protein pool in all three specimens, plasma, PBMCs, and brain, are similar from 12 to 25 months of age, again suggesting that circulating blood acetyl/total p53 may be an additional systemic marker for age-related changes in brain.

## DISCUSSION

Central to our findings is the observation that miRNA-34a expression in PBMCs and brain shows concordant steady increase from neonatal to old age, with levels of miR-34a in plasma showing a sharp rise from 4 to 25 months of age. This rapid gain of miR-34a in plasma is inversely related to SIRT1 abundance in this blood specimen, which declines precipitously from 4 to 25 months. However, in brain and PBMCs, this reciprocal expression between increased miR-34a and decreased SIRT1 is not observed until after 12 months of age. This pattern, starting at 4 months in plasma and 12 months in brain and PBMCs, is not observed with another major target of this microRNA, Bcl2, whose downward trend starts at neonatal time in plasma, while in brain and PBMCs it remains stable. In situ hybridization data and histochemical studies indicate the hippocampus as the venue of the most prominent local increase in expression level of miR-34a with age, and reciprocal decrease of SIRT1 in the same brain region in old mice [26]. Taken together, our results suggest that: 1. Increased levels of expression of miR-34a in plasma and PBMCs correspond to those observed in brain, with the former most dramatically preceding the latter two; and 2. the sharp increase in plasma miR-34a, and the decrease of SIRT1, starting at 4 months of age, may serve as noninvasive biomarkers for impending age-dependent brain decline at 12 months or later in mice.

The major difference between the two blood samples, PBMCs and plasma, is the source of their RNA and protein specimens. PBMCs largely consist of cells making up this component, including lymphocytes, monocytes, macrophages, etc., while plasma components are released from various tissues into circulating blood through secretion, exocytosis, cell-to-cell communication, and even cell death, including both apoptosis and necrosis [30]. Thus, in mammals, cell-free plasma RNA and protein may be true ‘foot-prints’ of the entire organism's health status. Obviously, changes in PBMC RNA and protein profiles follow the same age-dependent regulation as other organs, i.e. cell-type specific. Nevertheless, our observations suggest that increased levels of miR-34a in PBMCs and plasma may be a noninvasive biomarker reflecting changes in brain during aging, with increased levels of this microRNA in plasma potentially serving as an early biomarker for impending changes in the brain.

Functionally, miR-34a is well recognized as a tumor suppressor in brain and many other tissues; its absence is associated with neoplastic growth, including glioma and brain tumor [31], explaining the needed increase during early adult life for protection against neoplasms of many cell types, including those in the central nervous systems (CNS). However, its continuing increase presents a classical case of antagonistic pleiotropy, i.e. a genetic trait beneficial in early life but posing adverse consequences in later life. This is largely due to the fact that, as do many other miRs, miR-34a silences multiple targets, suppressing cell-cycle traverse genes such as cdks and cyclins; but its silencing action on SIRT1 may be the most detrimental [31-33]. Although the notion of a direct role for SIRT1 and its sisters in extending life span has come into question recently [34], they clearly suppress age-dependent pathologies, reducing diabetes, obesity, neurodegeneration, etc. [35]. Thus, miR-34a's continuing rise in late life may cause a reduction of SIRT1, a loss ill afforded by older organisms [35-37].

Complicating further the see-saw relationship between miR-34a and SIRT1 expression is the role of p53 as transcriptional activator for this microRNA's expression [33]. Activation of miR-34a is regulated via binding of acetylated p53, which in turn is controlled by deacetylation by mammalian SIRT1, thus forming a feedback loop [33]. At first glance, our results may suggest that this loop may be disrupted in older mouse brain and PBMCs, as well as in many other tissues with their ‘foot-print’ in the plasma, due to increased miR-34a, decreased Sirt1 levels, and increased levels of acetylated p53. However, a recent report by Lee, et al. [36] of activation of nuclear bile acid receptor, Farnesoid X Receptor (FXR), recruiting the Small Heterodimer Partner (SHP) to the p53 binding site in miR-34a's promoter region, and thus preventing this microRNA's activation and its downstream suppressing action on SIRT1, presents yet another layer of control. Abnormality of this positive feedback between FXR/SHP to decrease miR-34a and increase SIRT1 is observed in many age-related metabolic diseases [36]. Our finding of continuing rise of miR-34a with age suggests that either continuing acetyl-p53 increase overwhelming the SHP-binding or other putative factors involved, thus disable the FXR/SHP role in inhibiting this microRNA activation, and cause the absence of SIRT1 in many age-related metabolic diseases.

As noted above, among the various functional impacts of p53, from tumor suppression to induction of apoptosis, there emerges yet another vital role in regulating signaling networks through controlling miR-34a transcription activation. This adds to the complexity of how p53 governs life span determination; contrasting scenarios report high tumor incidence in p53 knockout mice, thus shortening life span, while p53 overexpression induces accelerated aging [38, 39]. Therefore, p53 regulation is another case of antagonistic pleiotropy, protective in the young but deleterious in older organisms. For example, in the context of regulating intertwining networks, p53 seems to participate in life extension as a downstream activator for apoptosis in the well-known IGF1/mTOR pathway, suppressing tumor development; but its continuous increase in older life may overwhelm SHP's binding to miR-34a and become the dominant activator for miR-34a, resulting in the SIRT1 loss described above. To further thicken the soup, in a large human population study of p53 polymorphism, the p53-P72 genotype is associated with reduced fertility and increased longevity [40]. In brain, the role of p53 is associated with cognitive robustness, by regulating glucose metabolism, as well as neuronal apoptosis, eliminating deleterious, damaged neurons to make room for neurogenesis [40]. Here, SIRT1 is involved in neuroprotective signaling, reducing the formation of β-amyloid, the pathogenic form of amyloid precursor protein (APP), one of the two main diagnostic histopathologies of AD, along with neurofibrillary tangles. Paradoxically, β-amyloid activates p53, which in turn activates miR-34a and suppresses SIRT1 [41], as one of the putative mechanisms underlying accelerated aging when this tumor-suppressing transcriptional factor is overexpressed. The complexities presented here in p53-dependent regulation, and how miR-34a participates in this puzzle for brain aging, will demand future system biology investigation to unravel their mysterious but fascinating roles in regulating aging and life span determination. Nevertheless, our finding of the continuous increase of both acetyl-p53 and miR-34a in blood and brain during aging suggests them as two noninvasive biomarkers for aging in the central nervous system.

Circulating microRNAs, specifically in plasma, may reflect particular disease states such as cancer, cardiovascular disorders, and even neuronal dysfunction [18, 29]. Increases of miR-34a in various tissues during aging are well recognized in our own work and that of others, from liver to brain, and even in autopsy brains and PBMCs of AD victims [13, 16]. Our previous report [13] documented increased expression of miR-34a in AD patients’ PBMCs, compared to NEC, associated with allelic inheritance of APOE4 [19, 42]. Although this systemic change in miRNA expression in PBMCs of AD patients is of prime significance, expression profiles in PBMCs may also indicate changes in the immune system in Alzheimer's disease [42]. This notion led to the recent suggestion that aberrant inflammation is an vital underlying AD pathogenesis, challenging the popular emphasis on amyloid plaques as the essential manifestation of the disease [43]. The kernel of this suggestion is the overexpression of cytokine interleukin-1 (IL-1) by microglia in the brain, thought to lead to neuronal deterioration in Alzheimer's disease [44]. Interestingly, upstream regulators for IL-1, i.e. IL-6 and IL-8, are secreted by senescent fibroblasts, associated with miR-146a/b, key microRNAs controlling inflammatory response [45]. Moreover, increased miR-146a is noted in AD brains and the brains of older mice, perhaps due to increased inflammatory response [46]. Linking the secretion from senescent fibroblasts to brain aging and neurodegeneration may suggest that heightened inflammatory response in aged animals and Alzheimer brain is a systemic manifestation involving all tissue types; plasma samples may be the best source to identify the factors involved. Future work using our same approach, i.e. baseline studies of the composition of this blood component, with miR-146a and others secreted by cells such as senescent fibroblasts, may yield more blood-based, age-dependent biomarkers in the category of increased inflammatory response.

Clearly, the present study is not comprehensive, covering all the miRNAs found in either PMBCs or plasma as biomarkers of brain aging, as noted above for miR-146a. However, our finding of miR-34a increasing monotonically with age, as a first example, demonstrates that circulating blood is a powerful and accessible window to visualize changes in vital organs which are not otherwise available, in the case of human studies during aging. Including our own work with Alzheimer's disease (AD), most human studies are limited by available resources and time required, and thus designed with a cross-sectional approach, comparing disease victims with age-matched controls, as reviewed by Provost, 2010 [47]. Longitudinal follow-up studies are rare and costly, and thus most microRNA studies for AD pathogenesis are limited to autopsy brain samples and animal models. Neither approach is ideal; the former is noted for its graveyard nature, imperfect neither for disease initiation nor progression, and animal models mostly use transgenic mice carrying human APP and/or Tau mutations mostly composed of familial AD polymorphisms as surrogates. The present work shows baseline concordant changes between PBMCs, plasma specimens, and brain, thus validating our human peripheral blood sample study with AD patients, pointing to circulating RNAs as an accessible and noninvasive biological source to detect changes in brain. In particular, changes in plasma microRNA profile may occur much earlier, seen here at 4 months, than the impending decline in brain. Our study presents the first example that circulating blood microRNAs, exemplified by miR-34a, may serve as biomarkers for brain aging. The RNA samples collected for this study will be an invaluable resource, when the entire repertoire of microRNA profiles from birth to old age in mice is obtained by future studies using deep sequencing; data obtained will serve as a baseline database for animal disease model studies in general, and as a possible surrogate for human studies as well, because of the cross-species conserved nature of this noncoding RNA species. The present study paves the way for the ultimate discovery of tissue-specific biomarkers in blood samples for inaccessible organs such as brain, and possibly even blood-based universal biomarkers for entire organismic aging.

## MATERIALS AND METHODS

### Animals and Tissue Collection

Mice of the C57/black 6 strain, from 2 days till 31 months old, were used in this study. For immunohistochemistry and in situ hybridization assays, brains of males 10 and 31 months old were used, while the other assays used 2 days old till 25 months (2, 7, 14, 20 and 28 days, 2, 4, 12, 18, and 25 months). All animal work was approved by institutional (University of Louisville) biosafety board protocol #05-001. Brain and blood specimens, including both peripheral blood mononuclear cells (PBMC) and plasma, were obtained from at least three mice of each age group.

### Processing blood for PBMC and plasma fractions

After collection, individual mouse blood specimens were layered onto Ficoll-Paque Plus solution (GE Healthcare, Piscataway, NJ), containing EDTA to prevent coagulation, and centrifuged for 30 minutes at 1,500 x g to separate the blood samples into four layers, the plasma in the upper layer the next PBMC-containing fraction, as the white layer, then the Ficoll-Paque plus solution with the red blood cells at the bottom. The plasma and PBMC fractions were then collected and stored at −80°C until further processing for RNA and protein isolation.

Snap-frozen coronal brain sections were homogenized with Trizol reagent (Invitrogen, Carlsbad, CA), followed by the total RNA isolation, in parallel with the isolated PBMC specimens, with the RNease Mini Kit (Qiagen, Valencia, CA). The isolated RNA fractions were then dissolved in RNase-free water and stored at −80°C until use. The quality of the isolates was determined by the Agilent 2100 Bioanalyzer with the Agilent RNA 6,000 Nano kit (Agilent Technologies, Foster City, CA) by the RNA integrity number (RIN); samples with values > 7 are of acceptable integrity.

The RNA fraction was isolated by adding 0.75 ml of Trizol LS reagent (Invitrogen, Carlsbad, CA) to 0.25 ml plasma, followed by incubation at room temperature for 5 min. To this solution, 0.2 ml of chloroform was added; after vigorous mixing, it was further incubated for 5 minutes at room temperature, before centrifugation at 15,000 x g for 15 min at 4°C, to obtain the RNA aqueous phase. This was collected, and after addition of 0.5 ml isopropanol (Sigma, St. Louis, MO), RNA was precipitated and collected by further centrifugation to a pellet, before washing with 75% alcohol and storage in RNase-free water. Since plasma contains little or no 28S or 18S RNA, the integrity of the isolated RNA was determined by the small RNA peak between 0 and 150 nt (Supplemental Figure 5C). Concentrations of RNA specimens isolated from brain, PBMC and plasma were determined by Nanodrop 2000 (Thermo Scientific, Wilmington, DE).

### Determination of microRNA expression levels

Primers specific for miR-34a and miR-196a were obtained from Applied Biosystems (Foster City, CA) to perform quantitative RT-PCR (qPCR), according to this vendor's protocol for TaqMan microRNA assays. Isolated total RNA fractions from brain, PBMC and plasma were initially processed for reverse transcription (RT) using two miRNA-specific primers (miR-34a: AB Assay IDs 000426; miR-196a: 241070_mat) to obtain their RT products, used subsequently for qPCR analysis on a 7500 Fast System Real-Time PCR cycler (Applied Biosystems). Small RNA 202 was used as control to calculate the expression levels of two microRNAs, miR-34a and -196a in isolated biological specimens. Numerical indices of these expression levels are expressed by the 1/ΔCT method, and obtain the values for individual microRNA after subtraction of the CT value for snoRNA202 (AB assay ID 001232, Applied Biosystems).

### Isolation of Protein fraction from brain, PBMCs and plasma

Sister blocks of brain coronal sections used for RNA isolation were used for protein extraction. In brief, brain specimens of ~100 μg were solubilized in 300 μl RIPA buffer before isolating the protein fraction, as described in our earlier report [16]. For the PBMC specimens, the organic phase after RNA isolation, as described above, was dialyzed on Spectra Pro 6 dialysis membranes (Spectrum Laboratories Inc., Rancho Dominguez, CA) in 0.3% SDS buffer at 4°C until the precipitate was completely dissolved, with further incubation in 0.1% SDS solution for an additional 24 hours. Plasma contains a huge amount of serum albumin and immunoglobulin (IgG); these were removed by the Vivapure anti-human albumin serum (HAS)/IgG kit (Sartorius Stedim Biotech GMbH, Göttingen, Germany). BCA protein assays from Pierce Biotechnology Inc (Rockford, IL) were used to determine the concentrations of the isolated protein samples from brain, PBMC and plasma.

### Immunoblotting for protein levels of SIRT1, Bcl2 and p53

SIRT1 and Bcl2, two main targets of miR-34a, and the activator of this microRNA, acetyl-p53, as well as the total p53 protein pool were selected for determination of their protein abundance in brain, PBMC and plasma. Twenty-five micrograms of isolated protein samples from these three specimens were used for SDS-PAGE; afterwards, the electrophoretically separated bands were transferred from the gels to nitrocellulose membranes (Schleicher & Schuell BioScience, Keene, NH). Identification and quantification of the three proteins of interest were performed by incubating the membranes with antibodies including rabbit anti-SIRT1 (1:500, 75435, Abcam Inc., Cambridge, MA), rabbit anti-Bcl-2 (1:100, 2870, Cell Signaling, Danvers, MA), rabbit anti-acetyl-p53 (1:500, 06-758, Upstate (Millipore), Billerica, MA), rabbit anti-p53 (1:500, SC-6243, Santa Cruz Inc., CA) and rabbit anti-β-actin (1:1000, 8226, Abcam Inc., Cambridge, MA) overnight at 4°C. β-actin was used as a loading control for PBMC and brain specimens, but not for plasma protein samples, since this protein's levels in plasma are not always stable. Instead, we selected a stable band with equal intensities across all lanes by Ponceau S staining [48]. Blots were developed following the method published previously [17], and levels of protein abundance were detected by the Enhanced Chemiluminescence (ECL) method, according to the manufacturer's instructions (Pierce Biotechnology, Rockford, IL). The intensities of bands on the ECL- developed films were quantified by densitometry using ImageQuant software (Molecular Dynamics Inc. Sunnyvale, CA).

### Localization of miR-34a distribution by in situ hybridization in brain

A microRNA 34a-specific locked nucleic acid (LNA) conjugated with digoxigenin (DIG), and a scrambled control, were purchased from Exiqon (Woburn, MA; miR-34a LNA probe: 38487-05; Scramble-miR LNA probe: 99004-01). These probes were incubated with the snap-frozen coronal brain sections, following our reported protocol [3]. Bound LNA probes were revealed by further incubating the sections with sheep anti-digoxigenin (DIG) antibody conjugated with alkaline phosphatase (AP) (1:2000; Roche, Indianapolis, IN) at 4°C overnight, followed by further detection of the DIG-AP substrate by nitrobluetetrazoliumchloride (NBT)/5-bromo-4-chloro-3-indolyl phosphate, toluidine salt (BCIP). Positive signal of the distribution of the LNA probes was detected by microscopic visualization, followed by image quantification with densitometry software (ImageQuant version 5.2, Molecular Dynamics).

Selected segments of images in the hippocampal and cortical regions were evaluated for miR-34a labeling intensity, compared to the scrambled control probe. Image quantification was performed using three animals per age group; the intensities of the LNA probes were obtained numerically from three repeats with three serial sections each. Thus, the final intensities for the two probes in each age group were obtained from three different animals, to control for inter-animal difference, and three repeats with three serial sections to control for inter-experiment variation. The scrambled control was used to normalize for background labeling noise.

### Immunohistochemistry localization of SIRT1 distribution in brain

Sister serial sections to those used above for in situ hybridization were processed to the determine SIRT1 distribution by initially fixing them with 4% paraformaldehyde (PFA), followed by blocking with 10% goat serum (Invitrogen, Carlsbad, CA) and incubation with rabbit anti-SIRT1 at 1:200 dilution (75435, Abcam Inc., Cambridge, MA) at 4°C overnight. The bound antibody was revealed by further incubation with goat anti-rabbit (1:400; Invitrogen) conjugated with Alexa fluor 594 for 40 minutes at 37°C. Antibody labeling was evaluated on a Zeiss fluorescence microscope (Carl Zeiss, Brighton, MI) and AxioVision Rel.4.6 imaging system; image analysis of the distribution and intensities of SIRT1 protein in brain specimens of the two age groups follow the same procedure described above.

### Data Analysis for statistical significance

The 1/ΔCT values of qPCR results for the expression levels of miR-34a, miR-196, and controls among different age groups were analyzed by Student's t tests, with p values < 0.05 as statistically significant difference between any two groups presented in all the graphs for this assay. ANOVA was used to analyze data from all age groups included in all the assays. Mean ± standard deviation (SD) was calculated for data obtained from quantitative evaluation for immunoblotting, in situ hybridization and immunofluorescence assays. Data analysis for these results proceeded as described for qPCR values. In all experiments, three different animals were used, to control for inter-animal variation; some of them performed three repeats, to control for inter-assay variance.

## SUPPLEMENTAL DATA

S-Figure 1 Age-dependent levels of miRNA-34a expression in various tissues in C57/B6 mice Panels (**A-B-C**) present box plot graphs of miR-34a expression in plasma, PBMCs and brain samples, along with graphs of the 1/delta CT trend. (n = 3; three different animals were used from each age group selected for the study.)

S-Figure 2 Age-dependent expression levels of miRNA-196a in various tissues of C57/B6 mice Panels (**A-B-C**) present box plot graphs of miR-196a expression in PBMCs, plasma and brain samples, along with graphs of the 1/delta CT trend. (n = 3; three different biological samples from each age group.)

S-Figure 3 Graphical presentation of age-dependent expression levels of miR-34a target gene SIRT1 in plasma, PBMCs and brain samples of C57/B6 mice Panels (**A, B, C**) show line graphs representing average intensity of SIRT1 expression, and trend lines depicting expression trends in various age groups, separated into different panels, with neonatal 2 to 28 days (d) in panel (a), and early adult from 2 months (m) to old age of 25 months in panel (b) in PBMCs, plasma and brain samples. (All graphs represent Mean ± SD; n = 3; three different biological samples from each age group.)

S-Figure 4 Age-dependent expression levels of miR-34a target gene Bcl-2 in blood and tissue samples of C57/B6 mice Panels (**A, B, C**) show average intensities of Bcl-2 expression, and trend lines depicting expression in various age groups, in plasma, brain and PBMCs samples. All graphs represent Mean ± SD; n = 3; three different biological samples from each age group.

S-Figure 5 RNA integrity analysis, using Agilent 2100 Bioanalyzer Panels (**A**) Table shows RNA Integrity Number (RIN) for PBMCs and brain samples across the age groups considered; (**B**) shows the representative graphical representation of 28s, 18s, and small RNA bands observed during RNA integrity analysis for PBMCs and brain samples; (**C**) shows the representative graphical representation of small RNA bands as a small peak, and absence of 28s and 18s RNA peaks, as expected, in plasma samples during RNA integrity analysis.

S-Table-1 Statistical analysis of miR-34a and miR-196a expression in blood and tissue samples of C57/B6 mice Tabular presentation of mean standard deviation calculation for miRs-34a and 196a in PBMCs, plasma and brain samples. (n = 3, three different biological samples from each age group)
