# Supplementary figures and images for: Circulatory miR-34a as an RNA-based, noninvasive biomarker for brain aging
Source: Aging (Albany NY). 2011 Oct 16;3(10):985–1002. doi: 10.18632/aging.100371 (PMC3229974; doi:10.18632/aging.100371)

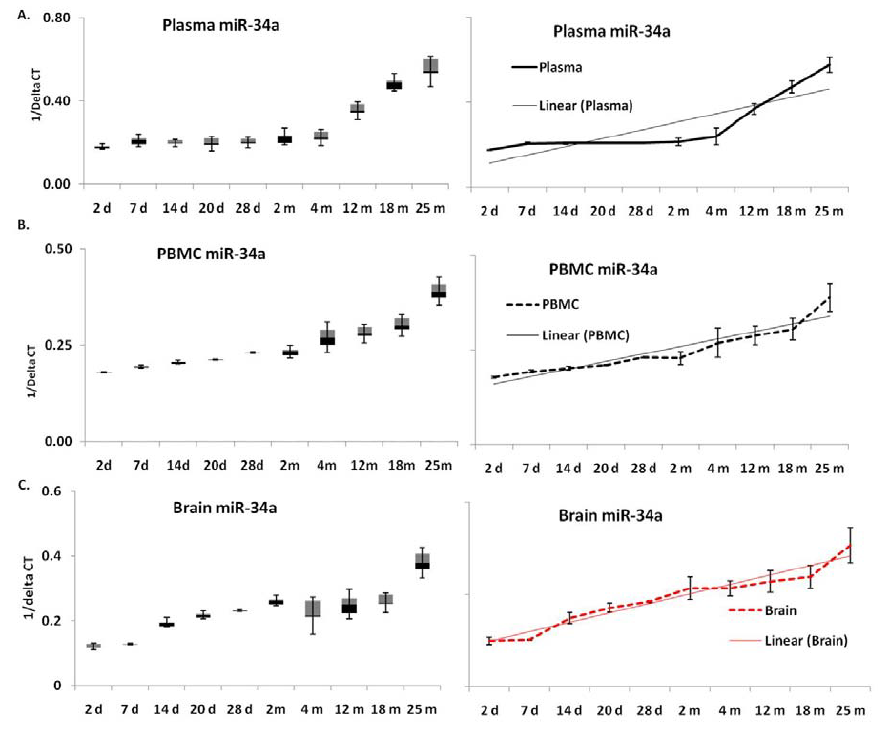

Supplement: S-Figure 1 — Panels (A-B-C) present box plot graphs of miR-34a expression in plasma, PBMCs and brain samples, along with graphs of the 1/delta CT trend. (n = 3; three different animals were used from each age group selected for the study.) [file aging-03-985-s001.tif]

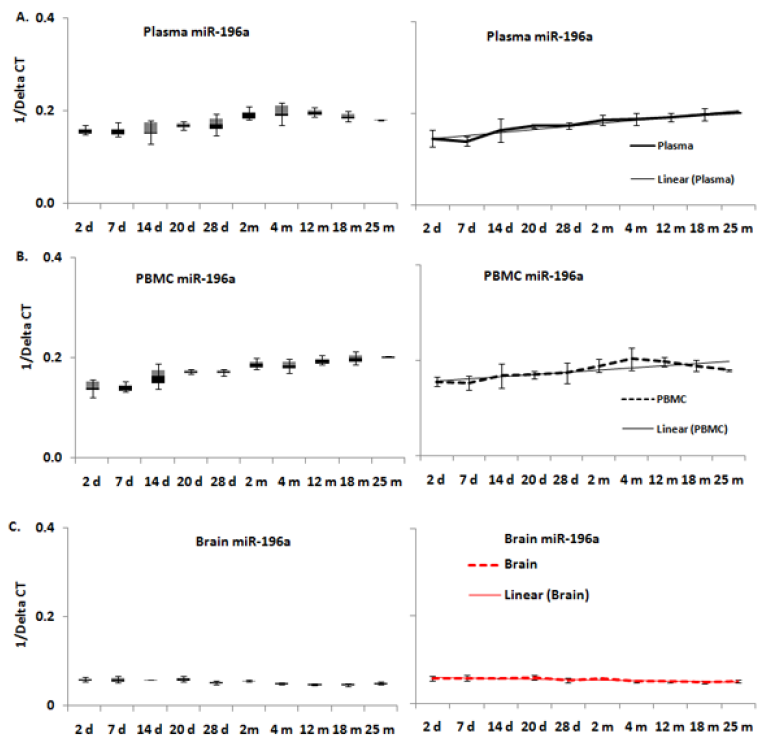

Supplement: S-Figure 2 — Panels (A-B-C) present box plot graphs of miR-196a expression in PBMCs, plasma and brain samples, along with graphs of the 1/delta CT trend. (n = 3; three different biological samples from each age group.) [file aging-03-985-s002.tif]

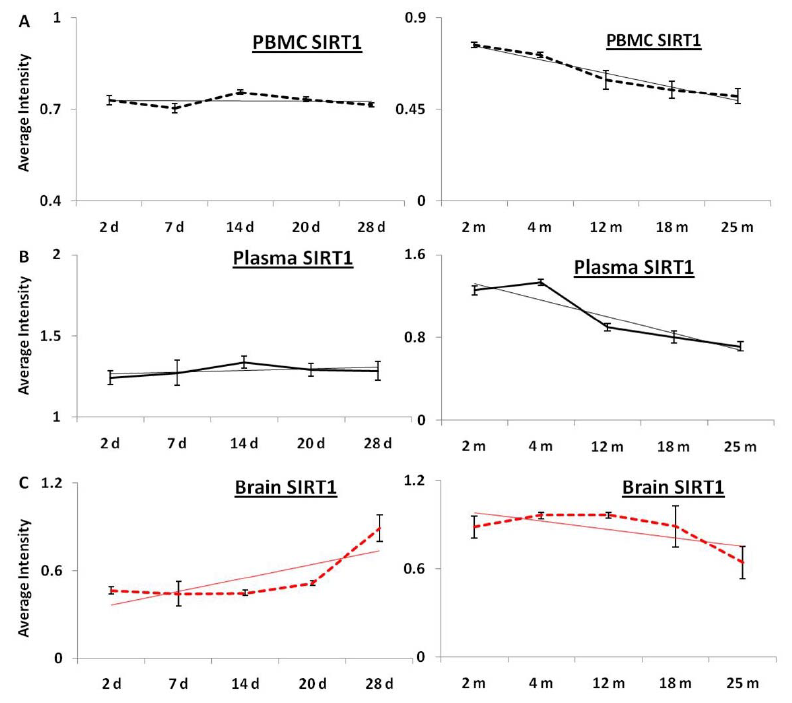

Supplement: S-Figure 3 — Panels (A, B, C) show line graphs representing average intensity of SIRT1 expression, and trend lines depicting expression trends in various age groups, separated into different panels, with neonatal 2 to 28 days (d) in panel (a), and early adult from 2 months (m) to old age of 25 months in panel (b) in PBMCs, plasma and brain samples. (All graphs represent Mean ± SD; n = 3; three different biological samples from each age group.) [file aging-03-985-s003.tif]

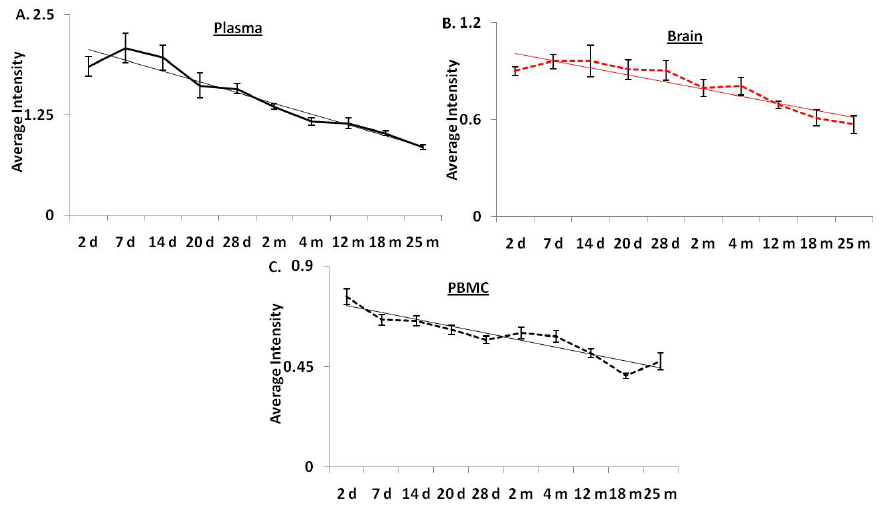

Supplement: S-Figure 4 — Panels (A, B, C) show average intensities of Bcl-2 expression, and trend lines depicting expression in various age groups, in plasma, brain and PBMCs samples. All graphs represent Mean ± SD; n = 3; three different biological samples from each age group. [file aging-03-985-s004.tif]

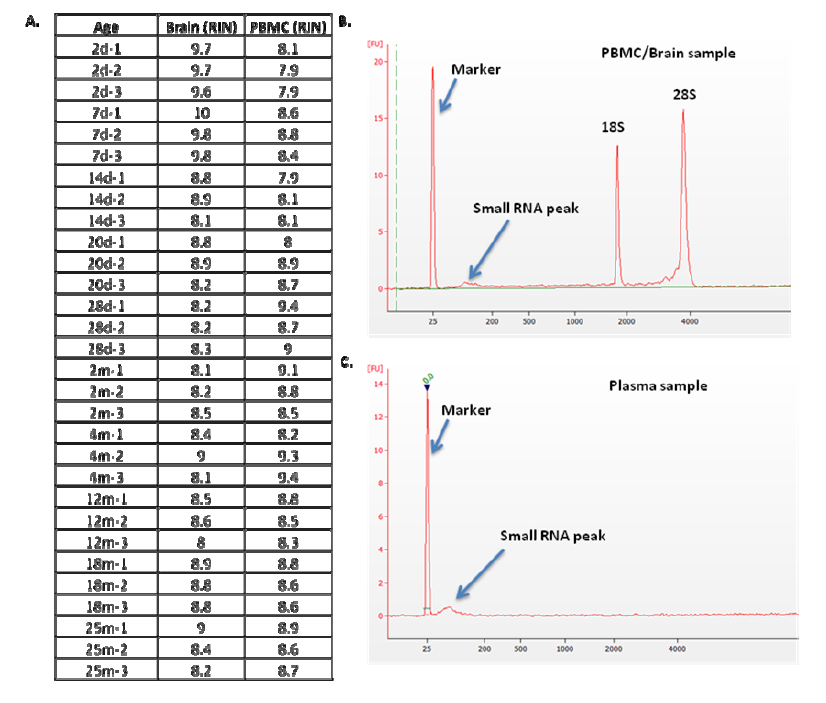

Supplement: S-Figure 5 — Panels (A) Table shows RNA Integrity Number (RIN) for PBMCs and brain samples across the age groups considered; (B) shows the representative graphical representation of 28s, 18s, and small RNA bands observed during RNA integrity analysis for PBMCs and brain samples; (C) shows the representative graphical representation of small RNA bands as a small peak, and absence of 28s and 18s RNA peaks, as expected, in plasma samples during RNA integrity analysis. [file aging-03-985-s005.tif]

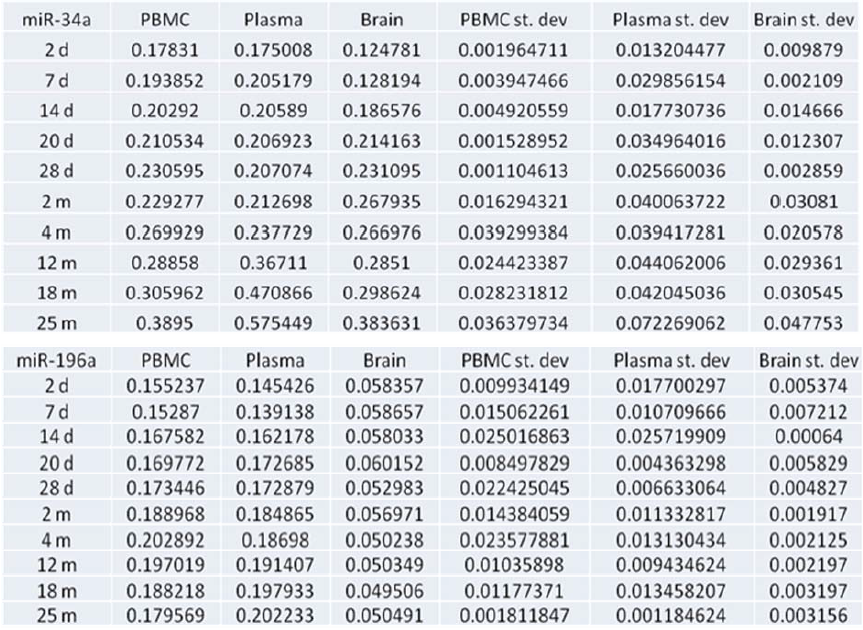

Supplement: S-Table-1 — Tabular presentation of mean standard deviation calculation for miRs-34a and 196a in PBMCs, plasma and brain samples. (n = 3, three different biological samples from each age group) [file aging-03-985-s006.tif]
